# Lithium carbonate-loaded polymeric nano-micelles for enhanced antitumor activity against NF1-associated malignant peripheral nerve sheath tumors *via* improved cellular uptake

**DOI:** 10.1039/d5na00789e

**Published:** 2026-03-27

**Authors:** Aiyun Yang, Jie Meng, Zhiqiang Zhu, Yuanfang Lu, Xiuwei Wang, Zhen Guan, Shen Li, Haiyan Xu, Zhichao Wang, Jianhua Wang

**Affiliations:** a Translational Medicine Laboratory, Capital Center for Children's Health, Capital Medical University, Capital Institute of Pediatrics Beijing 100020 China fywjh@163.com; b Institute of Basic Medical Sciences, Chinese Academy of Medical Sciences, School of Basic Medicine, Peking Union Medical College Beijing 100005 China xuhy@pumc.edu.cn; c Beijing Key Laboratory of Environmental & Viral Oncology, College of Life Science & Bioengineering, Beijing University of Technology Beijing 100124 China; d Department of Plastic and Reconstructive Surgery, Shanghai Ninth People's Hospital, Shanghai Jiao Tong University School of Medicine Shanghai 200011 China wangzhichao@sh9hospital.org.cn

## Abstract

Malignant peripheral nerve sheath tumors (MPNSTs) are fatal and highly aggressive soft tissue tumors that are associated with patients with neurofibromatosis type 1 (NF1). Traditional treatment methods have limited effects, so new treatment strategies need to be developed. Lithium carbonate (Li_2_CO_3_) is a type of psychotropic drug and has also been proven to play an important role in tumor treatment. Its nanoscale structure could achieve better therapeutic effects and reduce toxicity. In this study, we utilized MPNST cells (S462) derived from pediatric patients to develop polymeric nanomicelles loaded with Li_2_CO_3_ and determined their effects on the proliferation and apoptosis of MPNST cells and the related signaling mechanisms. The results indicated that LM significantly enhanced cellular uptake when exposed to the same concentration of extracellular Li_2_CO_3_. Free Li_2_CO_3_ inhibited cell proliferation in a concentration-dependent manner. Using a lower concentration could achieve the same or even stronger antitumor effect. The free drug and LM significantly increased the proportion of cells in the G1 and G2/M phases and inhibited the proportion of cells in the S phase (DNA synthesis phase). The effect of LM on the cell cycle was more significant compared with the free drug group. LM also significantly induced apoptosis in MPNST cells. The mechanism study revealed that LM increased the intracellular reactive oxygen species level and thereby inhibited the phosphorylation of ERK. After treatment with the antioxidant NAC, the expression of p-ERK increased, indicating that LM enhanced antitumor activity against MPNST cells by regulating the ROS–ERK signaling pathway. Our results suggested that encapsulating Li_2_CO_3_ in nanocarriers might reduce the required dosage and minimize adverse effects, which would provide a promising strategy for optimizing lithium-based therapies in MPNSTs.

## Abstract 2

Lithium carbonate-loaded polymeric nano-micelles for enhanced antitumor activity against NF1-associated malignant peripheral nerve sheath tumors *via* improved cellular uptake.

## Graphical abstract

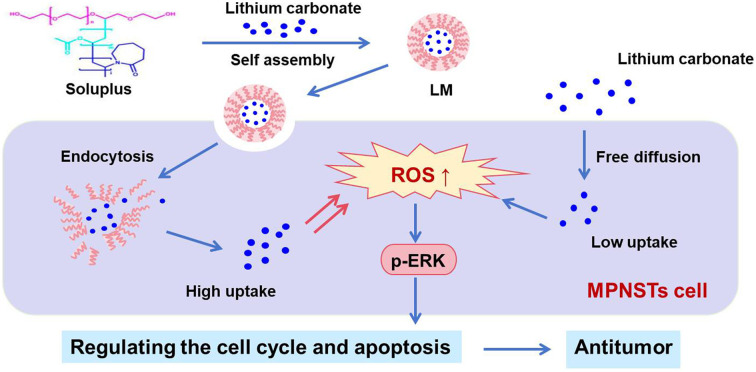

## 1. Introduction

Neurofibromatosis type 1 (NF1) in children is an autosomal dominant genetic disorder that occurs in childhood, involves multiple systems, and is accompanied by tumor susceptibility. The symptom spectrum is broad, and the main clinical manifestations are skin cafe-au-lait macules, neurofibromas, bone injuries and cognitive dysfunction.^1^ Patients with NF1 have a significantly increased risk of developing other tumors, and approximately 10% of them will develop malignant peripheral nerve sheath tumours (MPNSTs), with a high mortality rate.^2^ Malignant tumors are the most common cause of death for patients with this disease, and the average life expectancy is 10 to 15 years shorter than that of the general population.^3^ Resection surgery is the first-line treatment option for most NF1-related tumor complications. However, due to the location, size and invasiveness of the tumor, the surgery cannot remove all the tumors, and recurrence is prone to occur after the operation. The MEK inhibitor selumetinib is currently the only targeted drug for MPNSTs and has achieved certain therapeutic effects. Due to the immature development of various organs in children's bodies and the insufficient metabolism of the drug in children's bodies, it is prone to cause serious toxic and side effects, resulting in intolerance in children.^4^ Therefore, the treatment of MPNSTs still faces significant challenges.

Lithium carbonate (Li_2_CO_3_) is a psychotropic drug. In addition to its therapeutic applications, Li_2_CO_3_ has been proven to play an important role in anti-tumor treatment.^5^ Lactic acid in the tumor microenvironment causes more protons to be pumped into lysosomes through the lactylation of histones, thereby inhibiting the activity of tumor-reactive CD8^+^ T cells. Lithium carbonate enhances the anti-tumor immunity of CD8^+^ T cells by interfering with vacuolar ATPase, blocking lysosomal acidification, and shunting lactic acid from the nucleus to the mitochondria for oxidation.^6,7^ Other studies have shown that Li_2_CO_3_ inhibits the proliferation of different cervical cancer cells in a concentration-dependent manner, triggers DNA breaks in cervical cancer cells, and induces cell apoptosis through a caspase-independent pathway.^8^ In addition, when Li_2_CO_3_ is used in combination with other anti-tumor drugs, it enhances the differentiation-inducing effect on leukemia cells and targets leukemia stem cells.^9^ However, the pharmacokinetic characteristics of Li_2_CO_3_ show that it has a narrow therapeutic index, and its toxic dose is very close to the effective dose. Therefore, precise control over dosage and timing during lithium therapy is crucial to avoid adverse effects. Excessive Li_2_CO_3_ intake may lead to neurotoxicity or even irreversible nerve damage. Long-term use of Li_2_CO_3_ is also associated with a range of adverse reactions, such as thyroid dysfunction, kidney impairment, and cardiovascular complications.^10^ Therefore, it was necessary to seek methods to minimize the therapeutic dose of lithium while achieving its therapeutic effects, thereby reducing its toxic effects and improving patient outcomes.

Nanocarriers are materials at the nanoscale designed to deliver drugs, genes, or other biologically active molecules. They can enhance drug solubility, improve transport of poorly permeable water-soluble active ingredients, and traverse challenging biological barriers. In addition, nanocarriers also protect drugs from degradation in the external environment, prolonging their activity and maintaining stability. These properties of nanocarriers make them an attractive option for optimizing drug delivery and reducing toxic effects.^11,12^ Lithium salt nanoparticles have been applied in disease treatment. Injecting Li_2_CO_3_ nanoparticles into tumors attracts neutrophils and macrophages to the tumor site, leading to tumor cell necrosis.^13^ Nanocarriers also enable the use of lower drug doses while minimizing systemic toxicity. Studies have shown that lithium citrate and Li_2_CO_3_ nanoparticles inhibit the proliferation of liver cancer cells at lower concentrations, compared to the original form of lithium salts, with enhanced efficacy.^14,15^ It has been reported that chitosan-coated Li_2_CO_3_ nanoparticles facilitated a 1.3 times increase in cell proliferation in treated PC12 cells.^16^ These findings suggested that nanoparticles loaded with Li_2_CO_3_ might improve lithium's therapeutic efficacy and overcome its toxicity.

Although there is sufficient evidence showing the impact of Li_2_CO_3_ on different cancer models *in vitro*, its effect on MPNST cells is still lacking. In this study, we utilized an FDA-approved nanocarrier, Soluplus, to deliver Li_2_CO_3_ in the form of nanomicelles. The characterization of the nanomicelles was performed, and the antitumor effect and involved mechanisms were investigated. Our study suggested that encapsulating lithium salts in nanocarriers might reduce the required dosage and minimize adverse effects, which would provide a promising strategy for optimizing lithium-based therapies in NF1 treatment.

## 2. Materials and methods

### 2.1. Synthesis of LM

To prepare Li_2_CO_3_ nano-micelles (LM), 24 mg Soluplus (PCL–PVAc–PEG, BASF SE, Germany) and varying doses of Li_2_CO_3_ (Sigma-Aldrich, USA) were dispersed in 4 mL PBS and stirred at 800 rpm and 55 °C for 1 hour. After cooling to room temperature, the solution was centrifuged at 12 000 rpm for 10 minutes. The supernatant was carefully collected and passed through a 0.22 µm filter to ensure the removal of any remaining particulate matter. The filtered nanomicelle solution was stored at 4 °C for subsequent use in experiments.

For FITC-LM, 2 mg dithionic anhydride (Aladdin, China) and 1 mL LM solution were mixed with 4 mL activation buffer (pH 9–9.5 NaHCO_3_). 1 mg FITC (Sigma-Aldrich, USA) was dissolved in 10 mL sodium bicarbonate solution and added to LM solution, stirring continuously for 1 hour at room temperature. Then, 100 µL methanol was added and placed at room temperature for 10 min to terminate the activation reaction, and FITC-LM was obtained.

### 2.2. Characterization of LM

The hydrodynamic size and zeta potential of the LM were measured using a Zetasizer Nano ZS90 (Malvern, UK). The morphology of the LM was analyzed using a transmission electron microscope (TEM) (JEM-1400plus, Japan), to visualize the shape, size and structural integrity of the nanomicelles. Fourier transform infrared (FT-IR) spectroscopy was performed using a BRUKER Frontier (VERTEX 70, Germany) to analyze the chemical composition and encapsulation of Li_2_CO_3_ within the Soluplus nanomicelles. The stability of LM was observed over time and pictures were taken at different times.

### 2.3. Drug encapsulation efficiency and loading efficiency

To determine the efficiency of Li_2_CO_3_ encapsulation and loading in the LM, 1 mL of the LM solution was centrifuged at 12 000 rpm for 10 min to separate the free (unencapsulated) Li_2_CO_3_ from the nanomicelles. The supernatant was subjected to ultrasonic disruption for 10 min. The samples were transferred to a polytetrafluoroethylene crucible, 6 mL of nitric acid (Aladdin, China) was added, and the solution was heated to approximately 1 mL at 240 °C, on an electric heating plate. After cooling, the volume was adjusted to a fixed volume and analyzed using an ICP-MS instrument (iCAP PRO, Thermo Fisher, USA). Encapsulation efficiency = [(weight of drug)/(weight of the initial drug)] × 100%, which represents the percentage of Li_2_CO_3_ successfully encapsulated within the nanomicelles, reflecting the effectiveness of the encapsulation process. Loading efficiency = [(weight of drug)/(weight of the nanomicelles)] × 100%, which represents the percentage of Li_2_CO_3_ relative to the total weight of the nanomicelles, indicating the drug-carrying capacity of the nanocarrier.

### 2.4. Drug release assay

1 mL LM was sealed in a dialysis bag (molecular weight cut off: 8–14 KD, Millipore, MA, USA), immersed in PBS solution with pH 7.4 and 5.0, respectively, and maintained at 37 °C with 100 rpm shaking. At different time points (0.5, 2, 12, 24, 72, and 168 h), 3 mL was removed and the same volume of fresh PBS was added. ICP-MS (iCAP PRO, Thermo Fisher, USA) was used to detect the drug content at different time points and draw the drug release curve.

### 2.5. Cell culture

The human NF1-deficient malignant peripheral nerve sheath tumor cell line S462 (granted from Professor Zhichao Wang) was cultured with DMEM medium (Gibco, Grand Island, USA) containing 10% FBS (Gibco) and 1% penicillin/streptomycin (Gibco) at 37 °C and 5% CO_2_. The culture medium was replaced every 2–3 days. When the cells reached approximately 80% confluency, they were subcultured to prevent overgrowth.

### 2.6. Cellular uptake of LM

The S462 cells were cultured in glass bottom cell culture dishes with FITC-LM for 24 h and fixed with 4% polyformaldehyde (37 °C, 30 min). After incubation with DiI (1 : 500, cell membrane dye) for 10 min, slides were mounted with a DAPI-containing mounting medium (Zhongshan Goldenbridge, China) and observed under a confocal microscope (Leica, Germany).

### 2.7. Determination of the intracellular concentration of Li_2_CO_3_ by ICP-MS

S462 cells were seeded into a 6 cm culture dish at a density of 8 × 10^5^/dish and cultured overnight. Then, the cells were incubated with control, Li_2_CO_3_ (3 mmol L^−1^), or LM (3 mmol L^−1^) for 24 h, followed by washing with PBS twice to remove any extracellular Li_2_CO_3_ or LM. The cells were then digested and collected by centrifugation. The cell pellet was resuspended with 500 µL ddH_2_O and subjected to repeated frozen–thaw cycles at −80 °C to lyse the cells and release intracellular contents. The samples were analyzed using an ICP-MS instrument (iCAP PRO, Thermo Fisher, USA). A standard curve for Li_2_CO_3_ was prepared with concentrations ranging from 1 to 20 ppm, which exhibited a good linear relationship (*R*^2^ > 0.99). The concentration of Li_2_CO_3_ in all samples was calculated based on the standard curve.

### 2.8. Isolation of human peripheral blood mononuclear cells (PBMCs)

This study was performed in accordance with the Declaration of Helsinki and was approved by the Capital Institute of Pediatrics ethics committee, and written informed consent was obtained from all subjects. Fresh human peripheral venous blood (3 mL) anticoagulated with sodium citrate was diluted 1 : 1 with PBS. The diluted blood was gently mixed and carefully layered onto an equal volume of human lymphocyte separation medium. Centrifugation was then performed at 2000 rpm for 25 minutes. After centrifugation, the upper plasma and dilution layer were aspirated and discarded. The PBMC layer at the interface was carefully transferred to a new centrifuge tube. The cells were washed once with PBS and centrifuged at 1500 rpm for 5 minutes. Finally, the cell was resuspended in complete RPMI 1640 medium (Gibco) for subsequent experiments.

### 2.9. Cell viability assay

Cells in the logarithmic growth phase were collected and seeded into a 96-well plate at a density of 1.5 × 10^4^ cells per well. After overnight culture, the cells were treated with LM or free Li_2_CO_3_ for 24 h. Then, 10 µL CCK-8 solution (Dojindo, Japan) was added to each well and cultured for 4 h. The absorbance was measured at a wavelength of 450 nm using a Synergy H1 hybrid multifunction microplate reader (BioTek Instruments, USA).

### 2.10. Cell cycle assay

Cells were seeded into a 6-well plate at a density of 5 × 10^5^ cells per well and cultured overnight. The cells were then washed with PBS and starved with DMEM containing 2% FBS and 1% penicillin–streptomycin solution for 24 h. Then, S462 cells were treated with control medium, Soluplus (200 µg mL^−1^), Li_2_CO_3_ (3 mmol L^−1^), or LM (3 mmol L^−1^) for 24 h. After treatment, the cells were harvested and fixed in pre-cooled 70% ethanol at 4 °C overnight. Then, all samples were centrifuged and incubated in 100 µL of RNase (20 µg mL^−1^, Tiangen, China) at 37 °C for 30 min to degrade RNA and ensure specific DNA staining. Finally, the cells were stained with PI solution (50 µg mL^−1^, Solarbio, China) at room temperature for 15 min in the dark. The stained cells were analyzed using a flow cytometer. The average fluorescence intensity of the FL2 channel (PI emission) was detected. The cell cycle distribution was analyzed using FlowJo software.

### 2.11. Cell apoptosis analysis

S462 cells were seeded into a 24-well plate at a density of 1 × 10^5^ cells per well and cultured overnight. Then, the cells were treated with control medium, Soluplus (200 µg mL^−1^), Li_2_CO_3_ (3 mmol L^−1^), or LM (3 mmol L^−1^) for 24 h. After treatment, the cells were harvested and washed with PBS, incubated with Annexin V-FITC/PI (Dojindo Laboratories, Japan) at room temperature for 15 min, and the mean fluorescence intensity of FL1 and FL2 channels was detected by flow cytometry (Beckman, USA).

### 2.12. Detection of intracellular ROS

S462 cells were seeded into a 24-well plate at a density of 1 × 10^5^ cells per well. After overnight culture, the cells were incubated with Soluplus, Li_2_CO_3_, LM, or NAC (5 mM) for 24 h. The cells were washed twice with PBS, and a 10 µM 2′7′-dichlorodihydrofluorescein diacetate (DCFH-DA, Sigma-Aldrich, USA) probe was added to each well for 30 min. After incubation, the fluorescence intensity of DCF (indicative of ROS levels) was measured using a flow cytometer (Beckman, USA).

### 2.13. Western blot

Cells were lysed in RIPA buffer (Applygen, Beijing, China) containing PMSF (Solarbio, China) and protein phosphatase inhibitors (dilution, 1 : 1 : 100, Applygen, Beijing, China). Total protein concentration in the lysates was determined using a BCA protein assay kit (Thermo Fisher Scientific, USA). Equal amounts of protein were separated by sodium dodecyl sulfate polyacrylamide gel electrophoresis (SDS-PAGE) and transferred to polyvinylidene fluoride (PVDF) membranes (Merck Millipore, Germany). Next, the PVDF membranes were blocked with 5% skim milk in TBST for 1 h at room temperature and incubated with antibodies against ERK, p-ERK or actin (dilution, 1 : 1000, Cell Signaling Technology, USA) overnight at 4 °C. Then, the membranes were washed and incubated with a secondary antibody (dilution, 1 : 10 000, Abgent, USA) for 2 h at room temperature. Protein bands were visualized using a chemiluminescence analysis system (Tanon, China). The intensity of protein bands was quantified using ImageJ software. The expression levels of p-ERK were normalized to actin to account for variations in protein loading.

### 2.14. Statistical analysis

All quantitative data were expressed as mean ± standard deviation (SD). One-way analysis of variance (ANOVA) was used to assess the statistical significance of differences among experimental groups. Statistical analysis was performed using SPSS software (SPSS 30.0). As presented in the figures, *p* < 0.05 was considered statistically significant.

## 3. Results and discussion

### 3.1. Preparation and characterization of LM

**Fig. 1:**
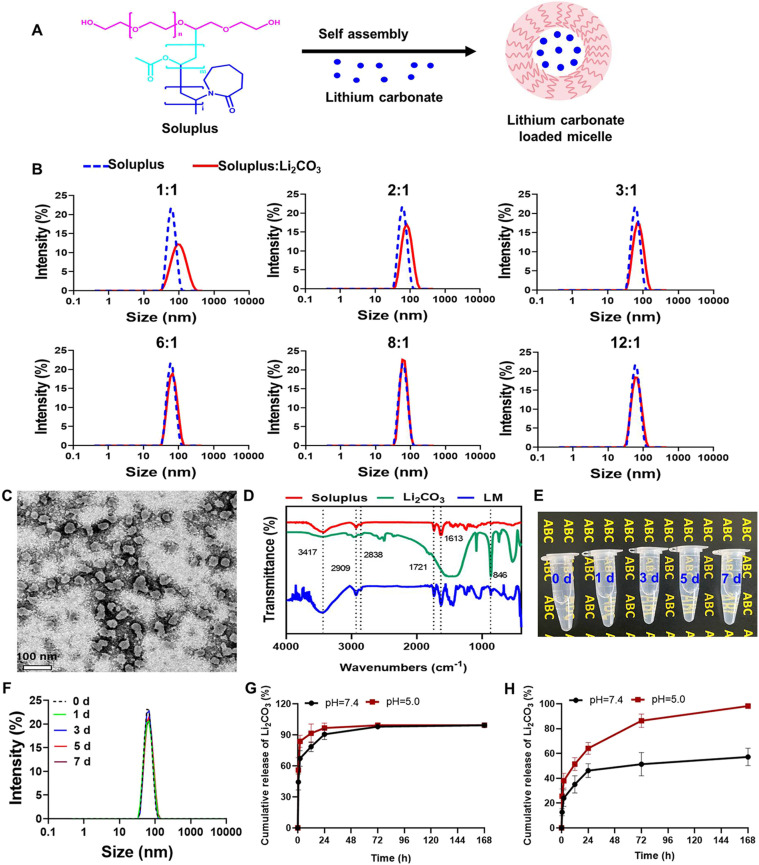
The preparation and characterization of LM. (A) Schematic of the preparation process of LM. (B) The hydrodynamic size of LM at different mass ratios of Soluplus and Li_2_CO_3_. (C) TEM image. Bar = 100 nm. (D) Fourier transform infrared spectroscopy of Soluplus, Li_2_CO_3_ and LM. (E and F) The appearance and the size of LM placed at different times. (G and H) Release profiles of Li_2_CO_3_ from the free drug or LM at varied pH. Data are shown as mean ± SD (*n* = 3).

Soluplus, an FDA-approved amphiphilic non-ionic polymer, forms nanomicelles in aqueous solutions and is widely used as a pharmaceutical excipient.^17^ As shown in Fig. 1A, Soluplus self-assembled into nanomicelles with a hydrophobic core and a hydrophilic shell. Lithium carbonate was encapsulated into the hydrophobic core of the micelles, forming LM. Soluplus and Li_2_CO_3_ were mixed at different mass ratios (1 : 1–12 : 1) to determine the optimal formulation. The hydrated particle size distribution of micelles in the solution was measured by dynamic light scattering (DLS). The hydrated particle size of the micelles followed a normal distribution (Fig. 1B). The results showed that the average hydrated particle size of empty Soluplus was about 60 nm. The hydrated particle size of LM was related to the mass ratio of Soluplus to Li_2_CO_3_. As the mass of Li_2_CO_3_ increased, the hydrated particle size of the LM increased, indicating successful loading of Li_2_CO_3_ into the micelles (Table 1). The polydispersity index (PDI) of the LM was around 0.10, indicating the formation of stable micelles with uniform particle size.^18^ The zeta potential of the empty Soluplus micelles is slightly negative (−1.25 mV). After adding lithium carbonate, the zeta potential showed a non-monotonic dependence on the ratio of the carrier to the drug (Table 1). This may be due to the combined effect of the carrier itself and the drug ions, as well as their mutual competition. In the case of high drug loading (1 : 1 ratio), a large number of lithium^+^ ions may partially neutralize the negative charge contributed by the adsorbed CO_3_^2−^ ions, resulting in a potential close to neutral. As the carrier ratio increased, the shielding effect of lithium^+^ ions weakened, making the negative charge generated by the adsorbed CO_3_^2−^ ions more significant, reaching a maximum at a 6 : 1 ratio. As the carrier ratio further increased, the absolute number of adsorbed ions decreased, causing the zeta potential to approach that of the blank micelles. These observations confirmed the successful loading of lithium carbonate and the dynamic regulation effect of the drug on the surface properties of the micelles. Based on stability and particle size, a mass ratio of 8 : 1 (Soluplus : Li_2_CO_3_) was the most stable and uniform LM formulation; thus, it was selected for subsequent experiments to evaluate the antitumor effects in MPNST cells.

**Table 1:** Size, PDI and zeta potential measured by DLS

Micelles	Mass ratio (Soluplus : Li_2_CO_3_)						
	1 : 0	1 : 1	2 : 1	3 : 1	6 : 1	8 : 1	12 : 1
Size (nm)	60.25 ± 0.23	90.77 ± 0.51	76.84 ± 0.38	69.57 ± 0.67	63.83 ± 0.17	62.13 ± 0.43	61.65 ± 0.47
PDI	0.03 ± 0.01	0.16 ± 0.01	0.12 ± 0.03	0.09 ± 0.01	0.06 ± 0.01	0.02 ± 0.01	0.05 ± 0.01
Zeta potential (mV)	−1.25 ± 0.12	0.16 ± 0.17	−0.44 ± 0.71	−0.86 ± 0.05	−1.44 ± 0.54	−1.18 ± 0.38	−0.86 ± 0.26

To confirm the structural and chemical properties of LM, we utilized TEM and FT-IR techniques. TEM images showed that LM exhibited a uniform spherical morphology with an average diameter of about 40 nm (Fig. 1C), which was different from the size measured by DLS. It is well known that the particle size obtained from DLS is the hydrated particle size based on a spherical model of nanoparticles.^19^ Therefore, the DLS results could be used to reflect the size homogeneity, while the exact size of the dry nanoparticles was detected by TEM. Soluplus is a triblock polymer composed of polyvinyl caprolactam–polyvinyl acetate–polyethylene glycol. The FT-IR analysis results (Fig. 1D) showed that the vibration of –OH can be determined at 3417 cm^−1^, and the stretching vibrations of –CH can be observed at 2909 cm^−1^ and 2838 cm^−1^. The characteristic peaks of the stretching vibrations of O=C–O and O=C–NH can be seen at 1721 cm^−1^ and 1613 cm^−1^. The bending vibration absorption peak of the carbonate ion can be observed at 846 cm^−1^. In the infrared absorption spectrum of LM, the corresponding characteristic absorption peaks of Soluplus and Li_2_CO_3_ can be detected, indicating that Li_2_CO_3_ has been successfully encapsulated within the micelles. Nanomicellar solutions usually appear transparent or semi-transparent, with weak light scattering; thus, the solution looks clear. When the solution remains stable, its transparency does not change significantly over time, so the background text can still be clearly seen after several days, indicating that no obvious aggregation or precipitation has occurred in the solution. The appearance (Fig. 1E) and size (Fig. 1F) of LM remained unchanged after being placed at room temperature for 7 days, indicating that LM had good stability. The encapsulation efficiency (EE%) of Li_2_CO_3_ in LM was 72.5%, meaning that 72.5% of the initial Li_2_CO_3_ was successfully encapsulated within the micelles. The drug loading efficiency (DL%) of Li_2_CO_3_ was 7.25%, indicating that 7.25% of the total weight of the nanomicelles consisted of Li_2_CO_3_. The high EE% and moderate DL% indicated efficient drug loading and encapsulation, which were important for achieving therapeutic efficacy with minimal drug loss. The release curves of Li_2_CO_3_ in free form and in LM were measured in PBS solutions with pH values of 7.4 and 5.0 (representing the typical pH values of cytoplasm and lysosomes, respectively). As shown in Fig. 1E and H, unlike free Li_2_CO_3_, which diffuses completely within hours, the LM demonstrated a pH-dependent sustained release behavior within 7 d. This would help evaluate formulation stability and sustained intracellular delivery. It indicated that once the LM was internalized and located in the acidic lysosomes, Li_2_CO_3_ could be rapidly released, thereby enhancing the intracellular drug concentration and efficacy.

### 3.2. LM increased the intracellular uptake of Li_2_CO_3_

**Fig. 2:**
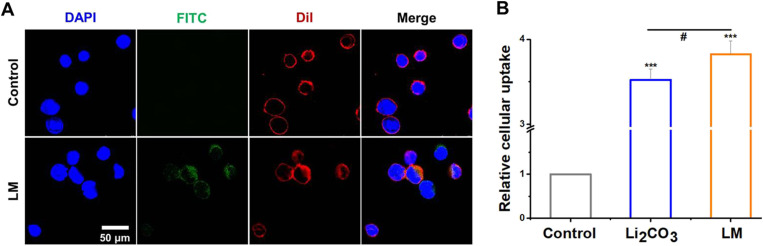
LM uptake by S462 cells. (A) Confocal images of the control (negative control, LM without FITC labeling) or FITC-LM after co-incubated with cells for 24 h. Scale bar = 50 µm. Cell nucleus (DAPI, blue), FITC-LM (FITC, green) and cell membrane (DiI, red). (B) The intracellular Li_2_CO_3_ content of cells after incubation with free Li_2_CO_3_ or LM at the same extracellular concentrations for 24 h. * represents the significant difference compared with the control. ***p < 0.001. ^#^ represents the significant difference between Li_2_CO_3_ and LM groups. ^#^*p* < 0.05.

The results of laser confocal microscopy showed that, compared with the negative control group treated with non-fluorescent-labeled micelles, FITC-labeled LM could be taken up by cells and was mainly distributed in the cytoplasm (Fig. 2A). In order to further evaluate the cellular uptake efficiency of LM, experiments were performed to compare the intracellular Li_2_CO_3_ content delivered by free Li_2_CO_3_ and LM containing the same concentration of Li_2_CO_3_. The intracellular lithium carbonate was quantified by measuring the lithium ion content in the cell lysate using ICP-MS. To eliminate the influence of cellular background and non-specific adsorption and to directly compare the intake differences among different groups, normalization was performed. The measured values of the Li_2_CO_3_ group and the LM group were respectively divided by the average value of the negative control group. The results demonstrated that compared with cells treated with Li_2_CO_3_ alone, LM significantly enhanced the intracellular uptake of Li_2_CO_3_ (Fig. 2B). These results indicated that LM significantly enhanced the cellular uptake of Li_2_CO_3_ when exposed to the same concentration of extracellular Li_2_CO_3_. The enhanced uptake of LM was likely due to the nanocarrier's ability to traverse cell membranes more efficiently than free Li_2_CO_3_. By increasing the intracellular concentration of Li_2_CO_3_, LM would enhance the therapeutic effects of Li_2_CO_3_ while minimizing the required dose and reducing potential toxic effects.

### 3.3. LM enhanced the antitumor activity against MPNST cells

**Fig. 3:**
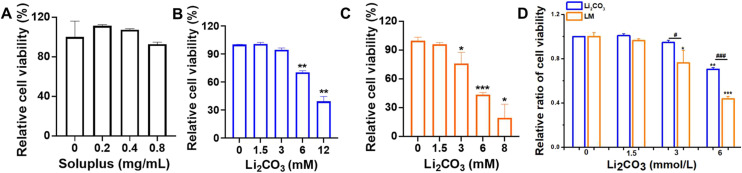
LM enhanced the antitumor activity against MPNST cells. The relative cell viability of (A) Soluplus, (B) Li_2_CO_3_ and (C) LM. (D) The relative cell viability of Li_2_CO_3_ and LM at the same extracellular concentration of Li_2_CO_3_. Data are shown as mean ± SD (*n* = 3). * represents the significant difference compared with the control. *p < 0.05, **p < 0.01, and ***p < 0.001. ^#^ represents the significant difference between Li_2_CO_3_ and LM groups in (B) and (C). ^#^*p* < 0.05 and ^###^*p* < 0.001.

By encapsulating more drugs in micelles, stronger effects were produced.^16,20^ To verify the antitumor effect of LM, S462 cells were incubated with Soluplus, LM and free Li_2_CO_3_ for 24 h, respectively, and cell viability was determined by CCK-8 assay. To exclude the influence of the nanocarrier itself, the cytotoxicity of Soluplus was examined. The results showed that even at the highest concentration used in the experiments (0.8 mg mL^−1^, which far exceeded the concentration of Soluplus in LM), Soluplus did not produce significant toxicity (Fig. 3A), suggesting that the observed effects of LM were due to the encapsulated Li_2_CO_3_ and not the nanocarrier. Free Li_2_CO_3_ inhibited the cell proliferation in a concentration-dependent manner, with pronounced effects observed above 6 mmol L^−1^ (Fig. 3B). Compared with Li_2_CO_3_, LM further enhanced the proliferation inhibition of S462 cells (Fig. 3C). When the LM was 3 mmol L^−1^, the cell activity was significantly reduced to 76.1%, while free Li_2_CO_3_ at 3 mmol L^−1^ had a relatively small effect on the cell activity, which was 94.6%. When the Li_2_CO_3_ concentration was 6 mmol L^−1^, the cell activities of free drugs and LM were 70% and 43.6%, respectively. When the concentration of Li_2_CO_3_ was 12 mmol L^−1^, the cell activity of the free drug was 39.3%, while the cell activity of LM decreased to 19.5% when the concentration was 8 mmol L^−1^. At concentrations of 1.5, 3, and 6 mmol L^−1^, LM decreased cell viability by 4.5%, 19.6%, and 38.1%, respectively (Fig. 3D). As noted in the Introduction, the clinical application of Li_2_CO_3_ is indeed limited by its systemic side effects, such as neurotoxicity and kidney damage.^10^ LM showed better efficacy at the cellular level than free Li_2_CO_3_, and this enhancement may be attributed to the change in the cellular uptake pathway. The free drugs mainly enter cells through passive diffusion and are easily expelled through transport proteins such as P-glycoprotein, which is a common mechanism of drug resistance.^21^ In contrast, LM is internalized through endocytosis, bypassing these efflux pumps, resulting in more effective intracellular drug accumulation. This was evidenced by its significantly enhanced cytotoxicity compared to free Li_2_CO_3_ at the same concentration (Fig. 3C and D). Given that many cancer cells have enhanced endocytosis,^22^ this mechanism may confer a certain degree of selectivity at the cellular level. Moreover, the nanoscale size of LM (approximately 60 nm) is suitable for taking advantage of enhanced permeability and retention (EPR) effects, which could promote its accumulation in tumor tissues and reduce systemic toxicity.^11^

**Fig. 4:**
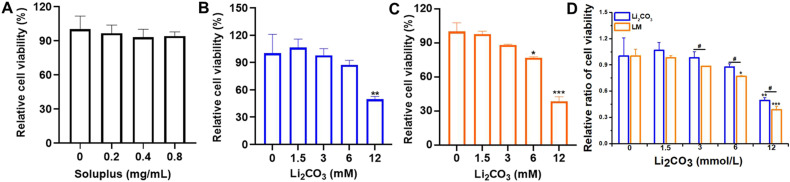
The relative cell viability of (A) Soluplus, (B) Li_2_CO_3_ and (C) LM in PBMCs. (D) The relative cell viability of Li_2_CO_3_ and LM at the same extracellular concentration of Li_2_CO_3_. Data are shown as mean ± SD (*n* = 3). * represents the significant difference compared with the control. *p < 0.05, **p < 0.01, and ***p < 0.001. ^#^ represents the significant difference between Li_2_CO_3_ and LM groups in (B) and (C). ^#^*p* < 0.05.

To evaluate the cytotoxicity of Li_2_CO_3_ and LMs on blood cells, the peripheral blood mononuclear cells (PBMCs) were isolated and treated with the same concentration of free Li_2_CO_3_ and LM as used in the MPNST cell experiments. As shown in Fig. 4, Soluplus alone showed no significant toxicity towards PBMCs at all tested concentrations. Both free Li_2_CO_3_ and LM reduce the viability of PBMCs in a dose-dependent manner. Moreover, at the same concentration, the cytotoxicity of LM is higher than that of Li_2_CO_3_. At concentrations of 3, 6, and 12 mmol L^−1^, the viabilities of PBMCs treated with Li_2_CO_3_ were 97.9%, 87.5%, and 49.5%, respectively, while those of the LM group were 88.3%, 76.8%, and 38.7%, respectively. Notably, the enhancement of cytotoxicity by LM was more pronounced in S462 tumor cells than in PBMCs. At a concentration of 6 mmol L^−1^, LM reduced the viability of S462 cells to 43.6%, compared to 76.8% for PBMCs. Furthermore, LM reduced PBMC viability to 38.7% at 12 mmol L^−1^, whereas a lower concentration of LM (8 mmol L^−1^) was sufficient to reduce S462 cell viability to 19.5%. These results indicated that LM exerted a more potent inhibitory effect on malignant MPNST cells than on normal blood cells under the same conditions. This differentiated effectiveness suggested that the LM system is expected to achieve strong anti-tumor effects with some hematological toxic reactions, with scope for improving the specificity in future studies.

### 3.4. LM regulated the cell cycle and apoptosis to enhance the antitumor activity against MPNST cells

**Fig. 5:**
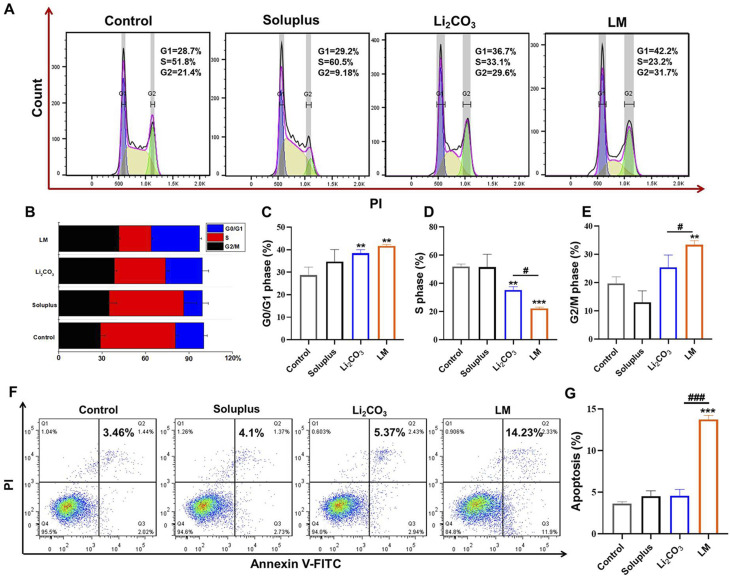
LM regulated the cell cycle and apoptosis to enhance the antitumor activity against MPNST cells. (A) Representative cell-cycle analysis performed with flow cytometry. (B–E) The proportions of G1, S and G2 phases in each group. (F) Representative scatter distribution for apoptosis after incubation with different groups for 24 h. (G) The quantification of apoptotic rate. Data are shown as mean ± SD (*n* = 3). * represents the significant difference compared with the control. **p < 0.01 and ***p < 0.001. ^#^ represents the significant difference between Li_2_CO_3_ and LM. ^#^*p* < 0.05 and ^###^*p* < 0.001.

Inducing cell cycle arrest is an important mechanism for inhibiting the proliferation of tumor cells.^23,24^ The results showed that both the free drug and LM significantly increased the proportion of cells in the G1 phase, inhibited the proportion of cells in the S phase (DNA synthesis phase), and significantly increased the proportion of cells in the G2/M phase. The effect of the LM group on the cell cycle was more significant compared with the free Li_2_CO_3_ group (Fig. 5A–E). Antitumor drugs also inhibited cell proliferation by inducing cell apoptosis.^25,26^ Flow cytometry results showed that Soluplus and 3 mM free Li_2_CO_3_ did not induce cell apoptosis, but 3 mM LM significantly induced MPNST cell apoptosis, with an apoptosis rate 3.75 times that of the control group (Fig. 5F and G). These results indicated that LM simultaneously regulated the cell cycle and inhibited cell apoptosis to suppress the proliferation of MPNST cells and exert anti-tumor activity. Free small molecule drugs like Li_2_CO_3_ entered cells mainly through diffusion, which was inefficient and susceptible to drug efflux. On the other hand, LM carried the drug into cells *via* the endocytosis pathway, allowing for more efficient intracellular delivery and resistance to efflux. This mechanism enabled LM to deliver a higher effective concentration of Li_2_CO_3_ to the cells, even at lower doses, thereby inhibiting cell proliferation while minimizing potential side effects.^27,28^ These results suggested that the use of LM as a drug delivery system inhibits the proliferative effects of Li_2_CO_3_ in MPNST cells, making it a promising approach for MPNST treatment.

### 3.5. LM enhanced antitumor activity against MPNST cells through regulating the ROS–ERK signaling pathway

**Fig. 6:**
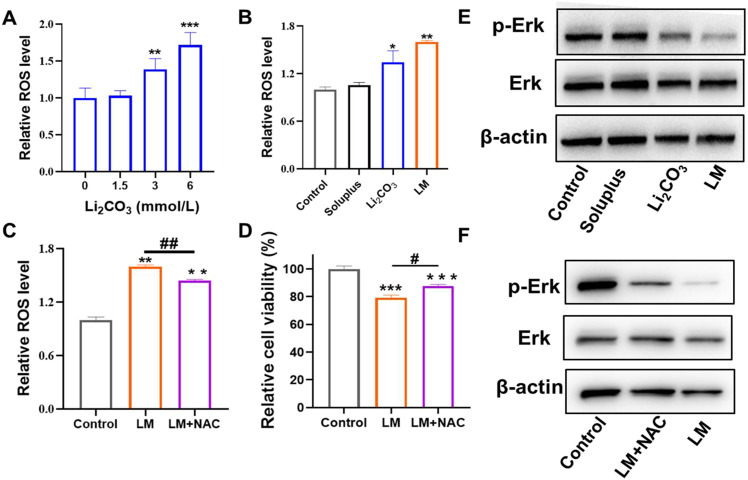
LM enhanced the antitumor activity against MPNST cells by inhibiting the p-ERK pathway *via* increased intracellular ROS levels. (A) The relative ROS levels in S462 cells treated with different doses of Li_2_CO_3_. (B) The relative ROS levels treated with Soluplus, Li_2_CO_3_ and LM for 24 h. The extracellular concentration of Li_2_CO_3_ was 3 mM for both Li_2_CO_3_ and LM groups. (C) Changes in intracellular ROS levels after treatment with the antioxidant NAC (5 mM). (D) The relative cell viability after treatment with the antioxidant NAC (5 mM). (E) Expression of p-ERK and total ERK in S462 cells after incubation with Soluplus, Li_2_CO_3_ and LM for 24 h. (F) Expression of p-ERK after treatment with the antioxidant NAC (5 mM). Data were shown as mean ± SD (*n* = 3). * represents the significant difference compared with the control. *p < 0.05, **p < 0.01, and ***p < 0.001. ^#^ represents the significant difference between LM and LM + NAC groups. ^#^*p* < 0.05 and ^##^*p* < 0.01.

Reactive oxygen species (ROS) are the main molecules produced during oxidative stress in the body.^29,30^ The imbalance of redox homeostasis is closely related to various diseases, especially tumors. Increasing the ROS level within tumor cells to cause oxidative stress and thereby inducing anti-tumor activity is considered an effective strategy for tumor treatment.^31^ The intracellular ROS levels of S462 were measured using a 2′, 7′-dichlorodihydrofluorescein diacetate (DCFH-DA) probe. The level of ROS increased gradually with the increase of Li_2_CO_3_ concentration (Fig. 6A). The LM group showed significantly higher ROS levels at the same concentration of extracellular Li_2_CO_3_ (3 mmol L^−1^) compared to the free drug group (Fig. 6B). In order to study the role of ROS in MPNST cell activity, the antioxidant *N*-acetylcysteine (NAC) was used to reduce intracellular ROS levels. It was found that treatment with NAC significantly decreased intracellular ROS levels (Fig. 6C), and this reduction in ROS was accompanied by a significantly increased cell proliferation (Fig. 6D), indicating that antitumor activity induced by LM was ROS-dependent. It has been reported that ROS regulated proliferation-related signaling pathways such as MAPKs and NF-κB signaling pathways.^32–34^ ERK, a member of the MAPK family, plays a key role in carrying signals from surface receptors to the nucleus. Phosphorylated ERK is translocated from the cytoplasm to the nucleus, where it activates transcription factors involved in cell proliferation.^35,36^ We observed decreased p-ERK expression in the free Li_2_CO_3_ treated group, while the LM group showed the lowest levels of p-ERK (Fig. 6E). When cells were treated with NAC, p-ERK levels increased (Fig. 6F), suggesting the association between ROS and ERK activation. By inhibiting the ERK signaling pathway through increased intracellular ROS, LM inhibited MPNST cell proliferation. This mechanistic insight highlighted the potential of LM as a novel approach to exert anti-tumor activity, through regulating the ROS–ERK signaling pathway.

## 4. Conclusions

In conclusion, self-assembled Li_2_CO_3_ nano-micelle (LM) enhanced antitumor activity against NF1-associated MPNSTs through the ROS/ERK pathway to affect the cell cycle and apoptosis *via* improved cellular uptake. Our study suggested that polymeric nanomicelles containing Li_2_CO_3_ had promising applications for treating MPNSTs. Further research would be needed to investigate the long-term safety and efficacy of LM *in vivo*.

## Author contributions

Aiyun Yang: conceptualization, methodology, investigation, data curation, writing – original draft, funding acquisition, and writing – review and editing. Jie Meng: investigation, resources, and formal analysis. Zhiqiang Zhu: investigation and formal analysis. Yuanfang Lu: methodology, investigation, and formal analysis. Xiuwei Wang, Zhen Guan, and Shen Li: methodology and investigation. Haiyan Xu and Zhichao Wang: conceptualization, supervision, resources, and writing – review and editing. Jianhua Wang: conceptualization, resources, funding acquisition, supervision, and writing – review and editing.

## Conflicts of interest

There are no conflicts to declare.

## Supplementary Material

NA-008-D5NA00789E-s001

NA-008-D5NA00789E-s002

## Data Availability

The data that support the findings of this study are available from the corresponding author upon reasonable request. Supplementary information (SI) is available. See DOI: https://doi.org/10.1039/d5na00789e.
