# Supplementary figures and images for: Lithium carbonate-loaded polymeric nano-micelles for enhanced antitumor activity against NF1-associated malignant peripheral nerve sheath tumors via improved cellular uptake
Source: Nanoscale Adv. 2026 Mar 27;8(8):2665–74. doi: 10.1039/d5na00789e (PMC13027219; doi:10.1039/d5na00789e)

Figure 6E

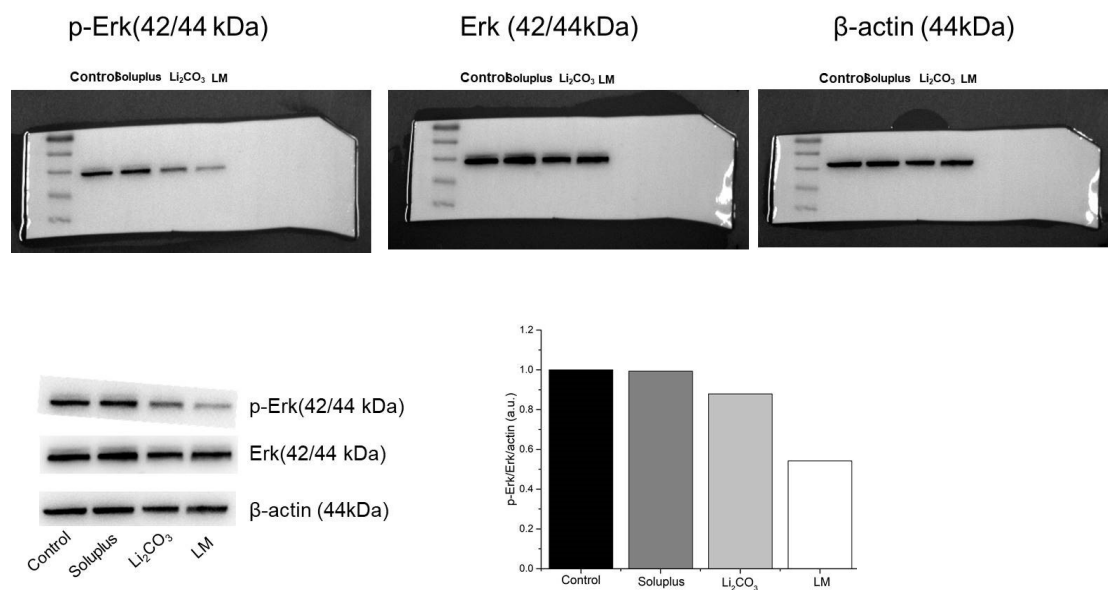

Figure 6F

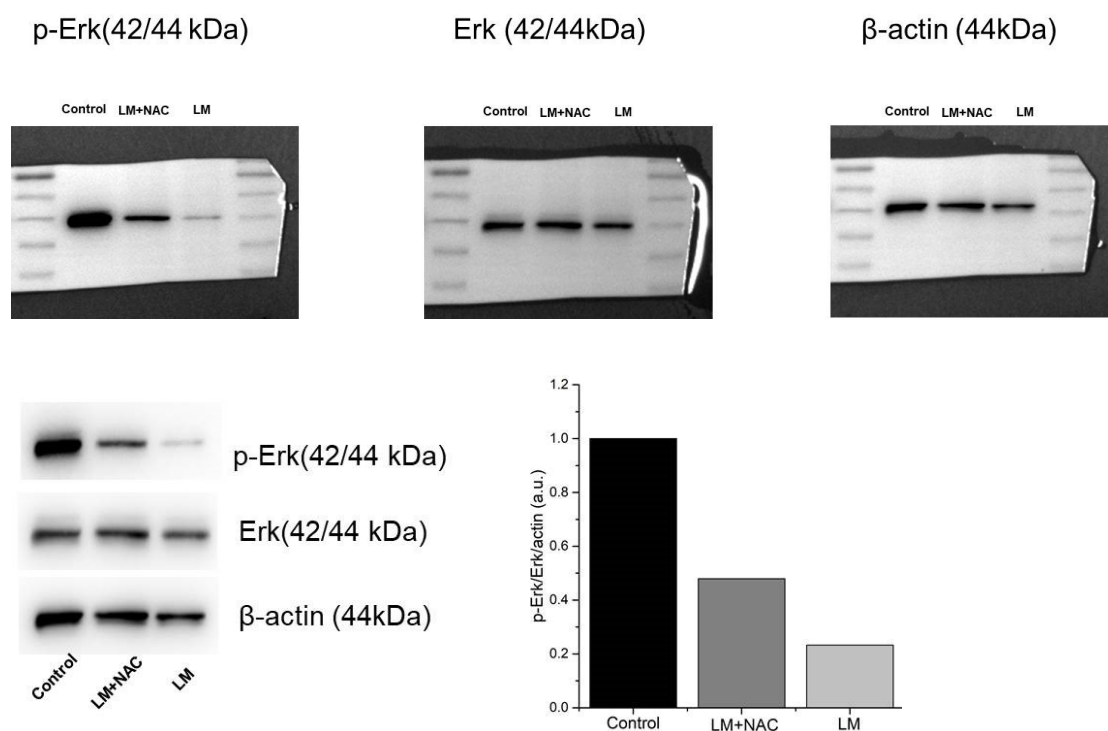

Supplement: NA-008-D5NA00789E-s001 [file NA-008-D5NA00789E-s001.pdf]

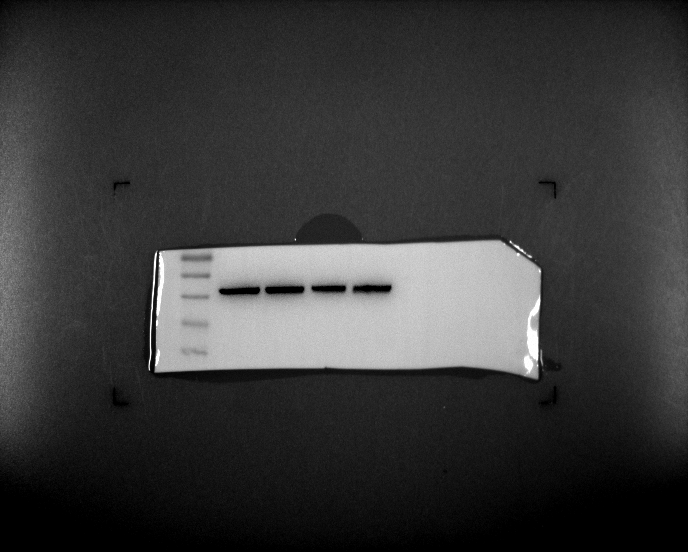

Supplement: NA-008-D5NA00789E-s002 [file NA-008-D5NA00789E-s002.zip › Supporting raw data-WB/Original data for WB-5E/actin marker.Tif]

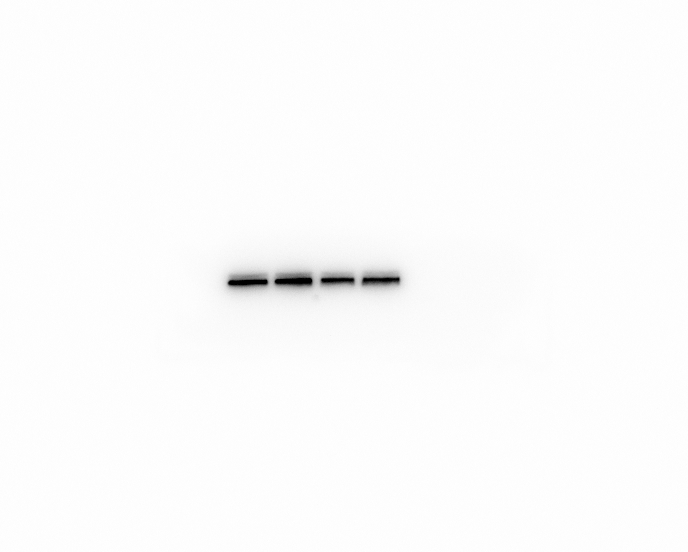

Supplement: NA-008-D5NA00789E-s002 [file NA-008-D5NA00789E-s002.zip › Supporting raw data-WB/Original data for WB-5E/erk.Tif]

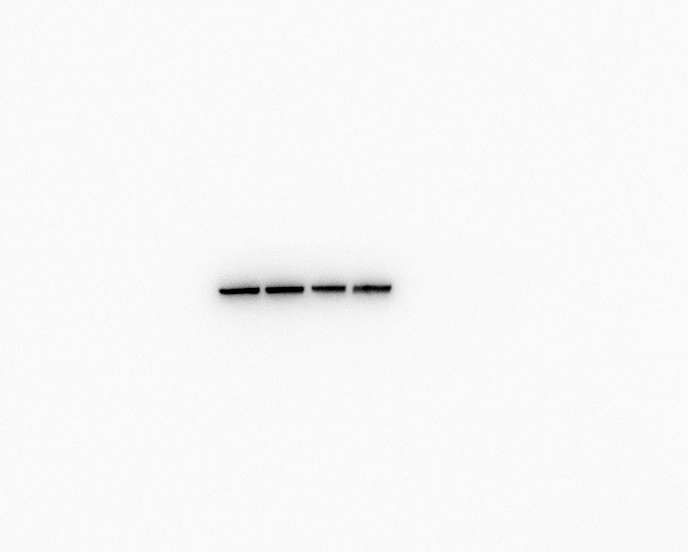

Supplement: NA-008-D5NA00789E-s002 [file NA-008-D5NA00789E-s002.zip › Supporting raw data-WB/Original data for WB-5E/actin.Tif]

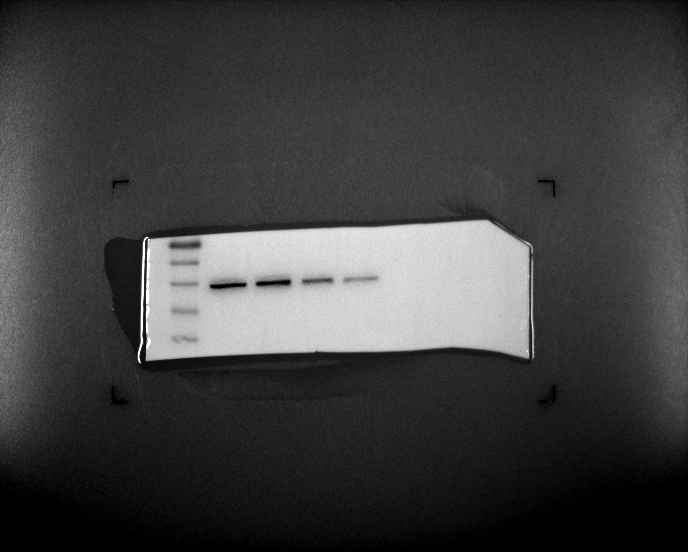

Supplement: NA-008-D5NA00789E-s002 [file NA-008-D5NA00789E-s002.zip › Supporting raw data-WB/Original data for WB-5E/p-erk marker.Tif]

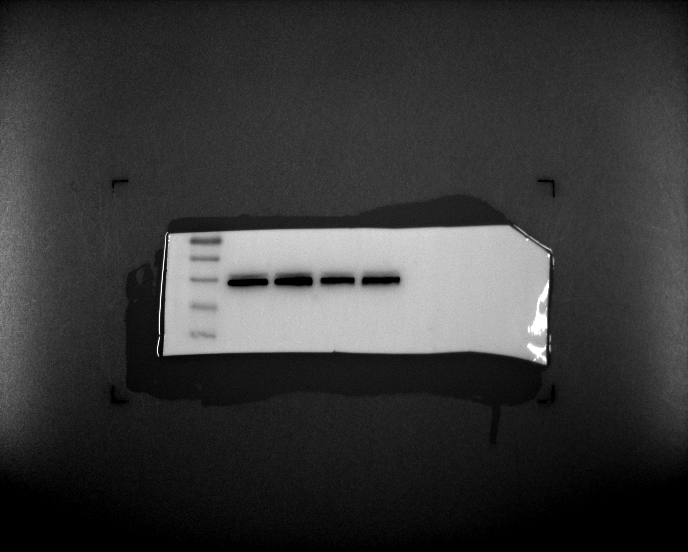

Supplement: NA-008-D5NA00789E-s002 [file NA-008-D5NA00789E-s002.zip › Supporting raw data-WB/Original data for WB-5E/erk marker.Tif]

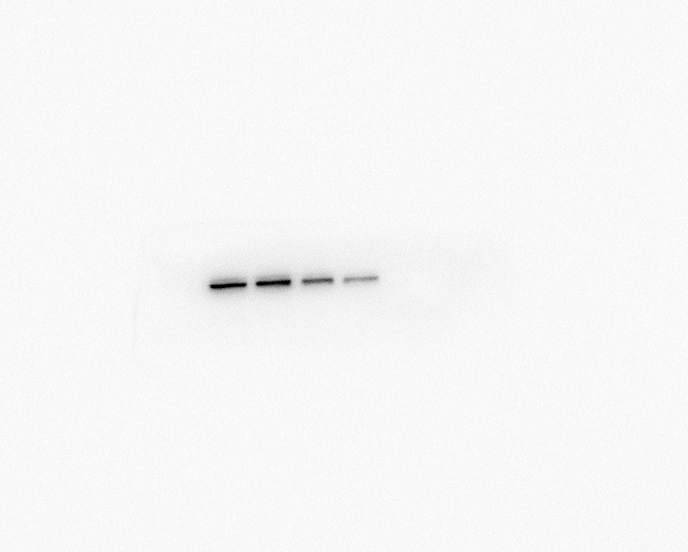

Supplement: NA-008-D5NA00789E-s002 [file NA-008-D5NA00789E-s002.zip › Supporting raw data-WB/Original data for WB-5E/p-erk.Tif]

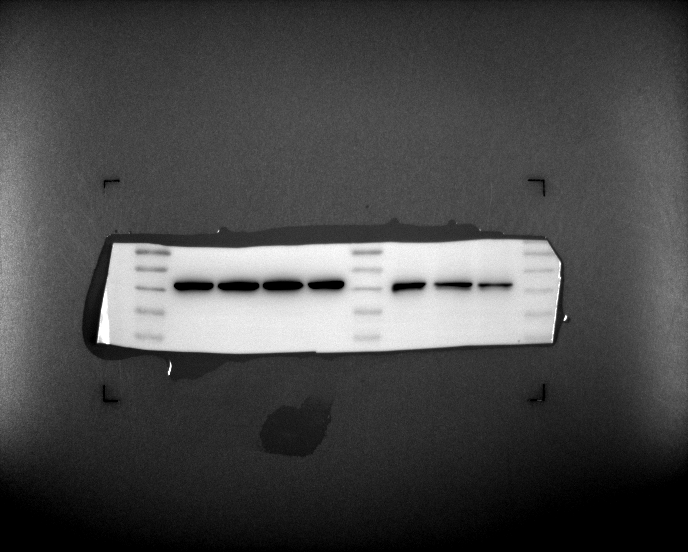

Supplement: NA-008-D5NA00789E-s002 [file NA-008-D5NA00789E-s002.zip › Supporting raw data-WB/Original data for WB-5F/actin-marker.Tif]

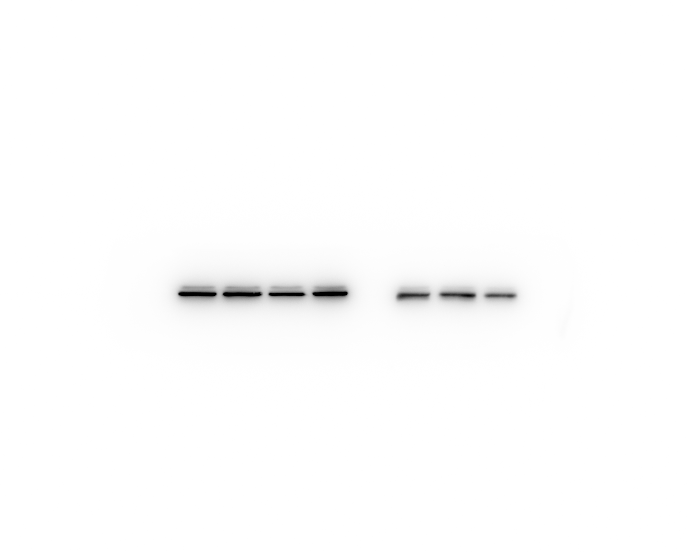

Supplement: NA-008-D5NA00789E-s002 [file NA-008-D5NA00789E-s002.zip › Supporting raw data-WB/Original data for WB-5F/erk.Tif]

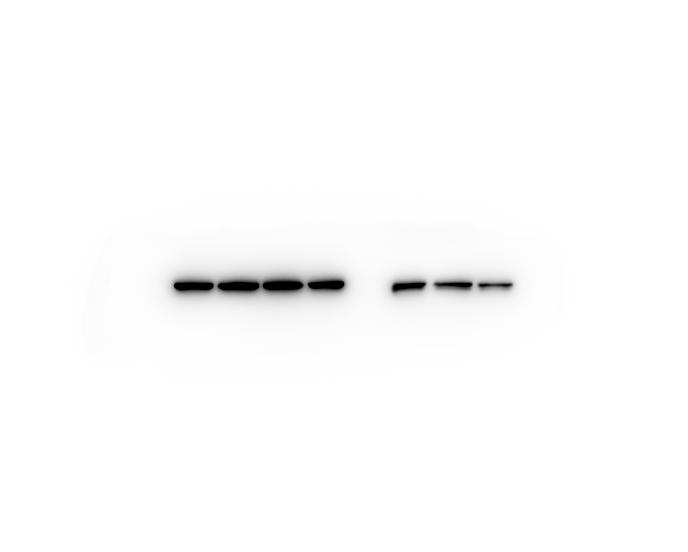

Supplement: NA-008-D5NA00789E-s002 [file NA-008-D5NA00789E-s002.zip › Supporting raw data-WB/Original data for WB-5F/actin.Tif]

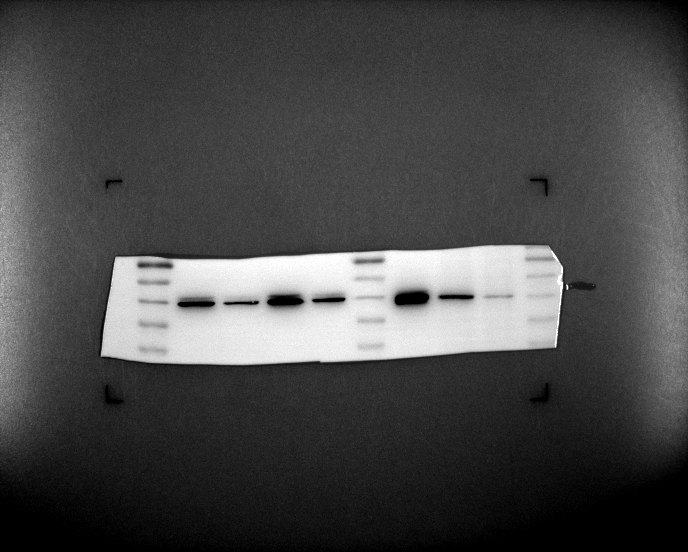

Supplement: NA-008-D5NA00789E-s002 [file NA-008-D5NA00789E-s002.zip › Supporting raw data-WB/Original data for WB-5F/p-erk marker.Tif]

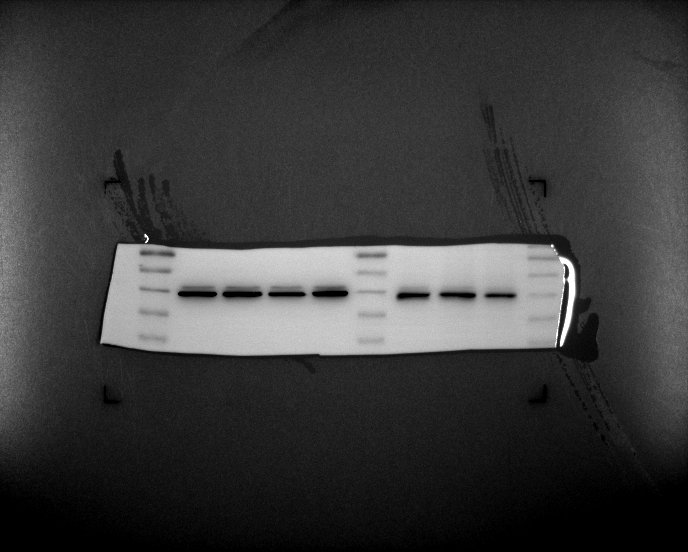

Supplement: NA-008-D5NA00789E-s002 [file NA-008-D5NA00789E-s002.zip › Supporting raw data-WB/Original data for WB-5F/erk-marker.Tif]

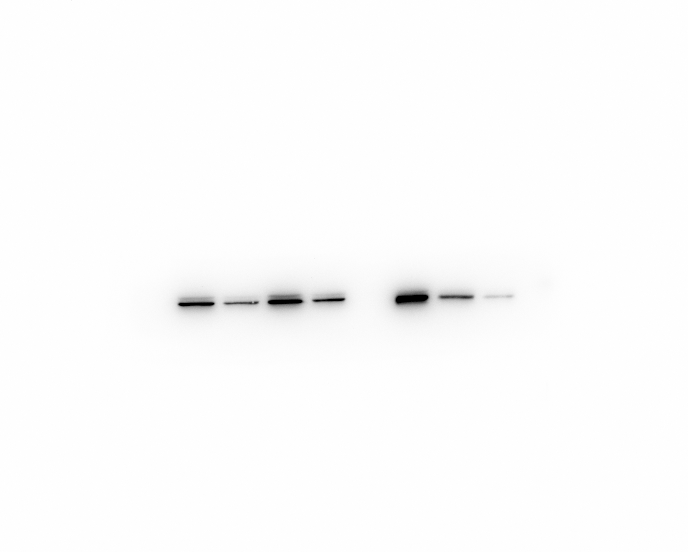

Supplement: NA-008-D5NA00789E-s002 [file NA-008-D5NA00789E-s002.zip › Supporting raw data-WB/Original data for WB-5F/p-erk.Tif]
