# Supplementary material for: Lithium carbonate-loaded polymeric nano-micelles for enhanced antitumor activity against NF1-associated malignant peripheral nerve sheath tumors via improved cellular uptake
Source: Nanoscale Adv. 2026 Mar 27;8(8):2665–74. doi: 10.1039/d5na00789e (PMC13027219; doi:10.1039/d5na00789e)

The markers on each band are all the same, the five protein ladder bands on the left, from top to bottom, represent 70, 55, 40, 35, and 25 KD respectively. The molecular weights of phosphorylated Erk, Erk and β-actin proteins are all 42 KD.


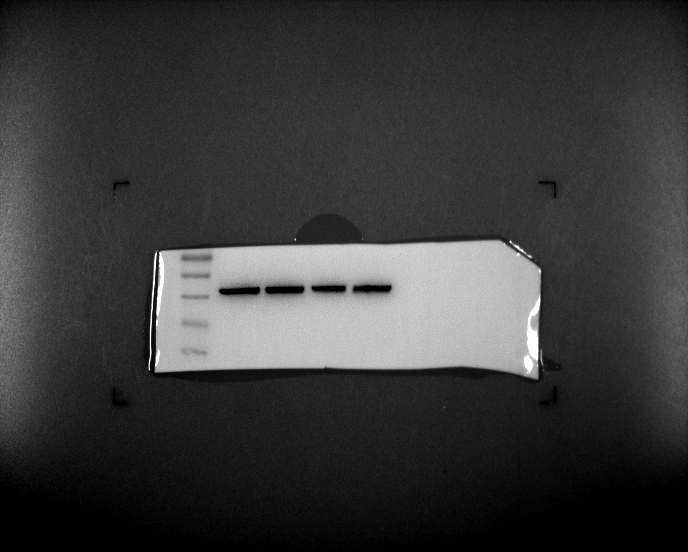


Catalogue numbers of the ladders used in the western blot experiments: 26616, Thermo Scientific


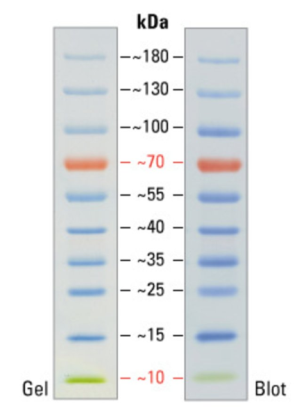

Supplement: NA-008-D5NA00789E-s002 [file NA-008-D5NA00789E-s002.zip › Supporting raw data-WB/weight markers and ladders explanation.docx]
